# Pressure for Pattern-Specific Intertypic Recombination between Sabin Polioviruses: Evolutionary Implications

**DOI:** 10.3390/v9110353

**Published:** 2017-11-22

**Authors:** Ekaterina Korotkova, Majid Laassri, Tatiana Zagorodnyaya, Svetlana Petrovskaya, Elvira Rodionova, Elena Cherkasova, Anatoly Gmyl, Olga E. Ivanova, Tatyana P. Eremeeva, Galina Y. Lipskaya, Vadim I. Agol, Konstantin Chumakov

**Affiliations:** 1 AN Belozersky Institute of Physical-Chemical Biology, MV Lomonosov Moscow State University, Moscow 119899, Russia; kel@sumail.ru (E.K.); galina.lipskaya@gmail.com (G.Y.L.); 2 Institute of Poliomyelitis and Viral Encephalitides of MP Chumakov Federal Scientific Center for Research and Development of Immune-and-Biological Products of Russian Academy of Sciences, Moscow 108819, Russia; apgmyl@mail.ru (A.G.); ivanova_oe@chumakovs.su (O.E.I.); poliom_ldms@mail.ru (T.P.E.); 3 US Food and Drug Administration, Silver Spring, MD 20993, USA; majid.laassri@fda.hhs.gov (M.L.); tatiana.zagorodnyaya@fda.hhs.gov (T.Z.); svetlana.petrovskaya@fda.hhs.gov (S.P.); rodionova.elvira@gmail.com (E.R.); 4 National Heart, Lung, and Blood Institute, National Institutes of Health, Bethesda, MD 20895, USA; cherkasovae@nhlbi.nih.gov; 5 IM Sechenov First Moscow State Medical University, Moscow 119991, Russia

**Keywords:** vaccine-derived polioviruses, attenuation, reversion, virulence, vaccine-induced adverse reactions

## Abstract

Complete genomic sequences of a non-redundant set of 70 recombinants between three serotypes of attenuated Sabin polioviruses as well as location (based on partial sequencing) of crossover sites of 28 additional recombinants were determined and compared with the previously published data. It is demonstrated that the genomes of Sabin viruses contain distinct strain-specific segments that are eliminated by recombination. The presumed low fitness of these segments could be linked to mutations acquired upon derivation of the vaccine strains and/or may have been present in wild-type parents of Sabin viruses. These “weak” segments contribute to the propensity of these viruses to recombine with each other and with other enteroviruses as well as determine the choice of crossover sites. The knowledge of location of such segments opens additional possibilities for the design of more genetically stable and/or more attenuated variants, i.e., candidates for new oral polio vaccines. The results also suggest that the genome of wild polioviruses, and, by generalization, of other RNA viruses, may harbor hidden low-fitness segments that can be readily eliminated only by recombination.

## 1. Introduction

Genetic recombination was demonstrated to occur in practically all viruses investigated in this respect [1], although its frequency in RNA viruses varies markedly, depending on the organization of the replicative machinery [2]. There is multiple experimental evidence that recombination may serve different evolutionary tasks, such as maintenance of the genome structure through purging deleterious mutations or conversely acquisition of beneficial or novel qualities. Nevertheless, the driving forces and biological consequences of specific instances of natural recombination in RNA viruses are rarely well understood. In this respect, the widespread occurrence of intertypic recombinants between the poliovirus vaccine strains is of special interest.

These strains were generated by Albert Sabin by selection from three serotypes of wild-type (wt) polioviruses [3]. Upon their derivation, they had accumulated various serotype-specific mutations, including those that were shown to be neurovirulence-attenuating [4]. Used as trivalent oral polio vaccine (OPV), these strains played a crucial role in the ongoing global campaign to eradicate poliomyelitis launched by the World Health Organization (WHO) in 1988 [5]. This very efficient vaccine has however some drawbacks. It is genetically unstable upon replication in vaccine recipients, readily losing attenuating mutations by reversions or compensatory changes [6,7,8], leading to (albeit very rarely) paralytic disease (vaccine-associated paralytic poliomyelitis; VAPP) in vaccinees or their contacts. Viruses excreted by healthy vaccine recipients or isolated from paralysis cases often turn out to be intertypic recombinants [9,10,11,12,13]. Moreover, the partially deattenuated variants can further evolve during long-term replication in the immunodeficient organisms or upon transmission through healthy populations, giving rise to more diverged, so called vaccine-derived polioviruses (VDPV), able to induce not only sporadic cases of poliomyelitis but also to cause more or less large outbreaks of the disease [14]. These VDPV are often recombinants either between different serotypes of Sabin viruses (or between Sabin and wt polioviruses) or between Sabin viruses and non-polio enteroviruses [15]. The specific reason(s) for the obvious propensity of Sabin viruses to recombine with suitable partners is poorly, if at all, understood.

Aiming to understand these reasons, in this study we have analyzed some aspects of a unique natural mega-experiment when a mixture of three well-studied and related viruses (the trivalent OPV) was given to billions of people and a plethora of progeny isolates were available for investigation. Specifically, we have sequenced, fully or partially, 98 genomes of intertypic poliovirus recombinants and analyzed these data together with the previously published results to infer common patterns of organization of the recombinant genomes. As a result, some important conclusions have emerged relevant not only for the understanding of the nature of Sabin viruses and their penchant for recombination but also of some general issues of biology of RNA viruses.

## 2. Materials and Methods

Virus strains derived from Sabin polioviruses were mainly collected in Russia and countries of the former Soviet Union from patients with acute flaccid paralysis (AFP), patients with other diseases as well as from healthy individuals and environment. Isolation, identification and intratypic characterization were performed according to the standard WHO polio surveillance protocol [16].

Nucleotide sequences were determined by full-genome deep sequencing as follows. Cell culture supernatants were treated with micrococcal nuclease to digest free nucleic acids, and RNA was isolated by RNeasy kit (Qiagen, Germantown, MD, USA). It was then fragmented by using focused ultrasonicator (Covaris, Woburn, MA, USA) to produce 200–400 nt-long molecules. cDNA was prepared by reverse transcription using Superscript III (Invitrogen, Waltham, MA, USA). Illumina adapters with index oligonucleotides (barcodes) were added and the resulting DNA library was amplified by PCR. Sequencing was done using Illumina Miseq in multiplex format with 10–12 barcoded samples analyzed in one run. For most samples treated this way, poliovirus-specific sequence reads represented the majority of all reads (50–95%), and the depth of coverage was between 10,000 and 200,000 reads per genomic position. All new nucleotide sequences were deposited in GenBank (accession numbers MG212427-MG212495).

Bioinformatic analysis was performed using two in-house software packages: ‘swarm’ program running on MacPro (Apple, Cuppertino, CA, USA) computers in UNIX (Darwin) environment, and cloud-based HIVE (highly-integrated virtual environment) system [17]. The analysis pipeline included the following steps. First, raw sequence data were pre-screened to eliminate low-quality reads and to remove adapter and index sequences. Next, sequence reads were aligned (mapped) to a curated database of 500 full-length reference sequences representing the entire spectrum of human enteroviruses. Recombination patterns were analyzed using Simplot algorithm [18]. The exact positions of crossovers were determined by comparisons of the sequences of recombinant RNAs with the relevant regions of their parental genomes of Sabin viruses with the help of ClustalW [19].

The location of crossovers in some recombinants investigated years ago and reported for the first time here were determined as follows. Viral RNA was extracted from the cultural medium of virus-infected cells with the Trizol reagent (Life Technologies, Washington, District of Columbia, USA) and reverse transcribed with avian myeloblastosis virus reverse transcriptase (Promega) using random hexamer primers (Boehringer Ingelheim, Ingelheim am Rhein, Germany). Approximate positions of the crossover were mapped by using the restriction fragment length polymorphism (RFLP) assay [20,21]. To this end, PCR amplification of several regions of the reverse transcripts was performed, and DNA products thus obtained were treated with restriction endonucleases chosen to generate unique electrophoretic profiles for each Sabin serotype. The inheritance of genomic regions from different parents (i.e., the recombinant nature) and the approximate positions of the crossovers could be thus deduced. To determine their precise location, the regions suspected to contain crossovers were PCR-amplified by using appropriate primers and sequenced manually [22] or by using ABI Prism 310 genetic analyzer (Applied Biosystems, Carlsbad, CA, USA).

## 3. Results and Discussion

### 3.1. Set of the Viruses Investigated

The set of intertypic recombinants between the Sabin vaccine polioviruses analyzed here, 189 samples, included 98 viruses collected mostly in Russia and countries of the former Soviet Union since the 1980s, whose genomic crossover sites were determined in this study either by full genome deep sequencing (70 samples, Table S1) or partial genome sequencing (28 samples, Table S2), as well as 91 isolates whose full (Table S3) or partial (Table S4) sequences were previously reported by us and other investigators. Over half of the samples (55%) were isolated from the acute flaccid paralysis (AFP) cases (Table 1). In a few cases, different recombinants were isolated from the same patient. The collection also included isolates from patients with other diagnoses, from healthy persons, and from sewage; the origin of 14 isolates is unknown. Fourteen, 78, and 97 viruses were of serotype 1, 2, and 3, respectively. No effort was made to select particular serotypes; therefore, relative numbers of recombinants of distinct serotypes in this dataset roughly reflects the frequency of their isolation by other authors, with type 2 and 3 recombinants being the most abundant [12,23].

As judged by the level of divergence of the recombinant’s capsid protein VP1-coding sequences from those of their vaccine parents, recombination generally occurred early in the evolution of the vaccine strains. Indeed, out of 107 genomes investigated in this respect, 87 could be classified, by using official criteria [24], as Sabin-like, i.e., “young” viruses (Table 1). On the other hand, there were several examples, when the “ages” of segments of a given virus originating from different parents appeared to markedly differ, suggesting the possibility of late recombination as well. Thus, the multipartite recombinant 13688 (Table S1) of structure S1-S3-S2-S1-S2-S3, having only 2 replacements (0.05%) in the proximal 3840 nucleotides derived from serotype 1, exhibited 2.2%, 1.3%, 4%, 0.8%, and 1.2% mutations in the successive segments, respectively.

### 3.2. Serotype-Specific Patterns of Recombination

**Figure 1:**
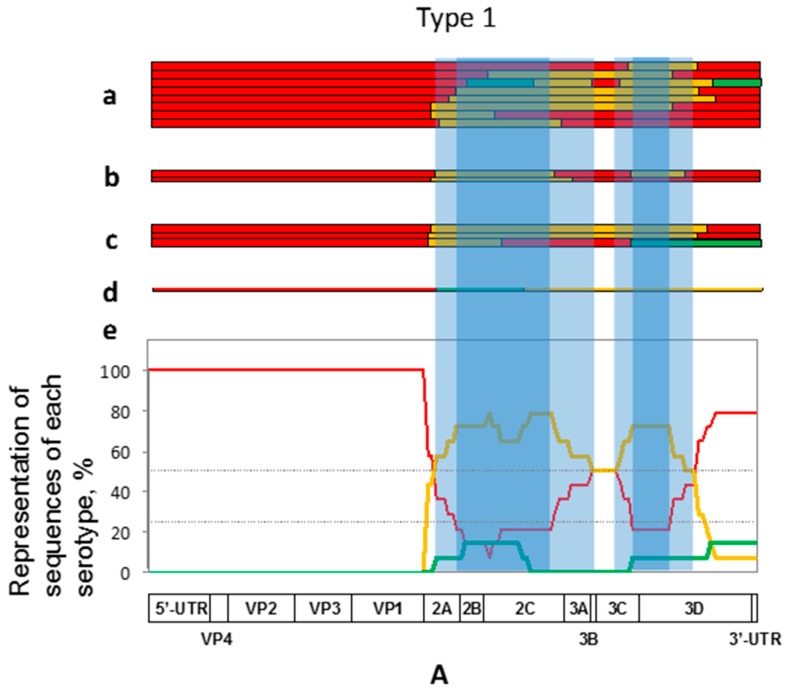

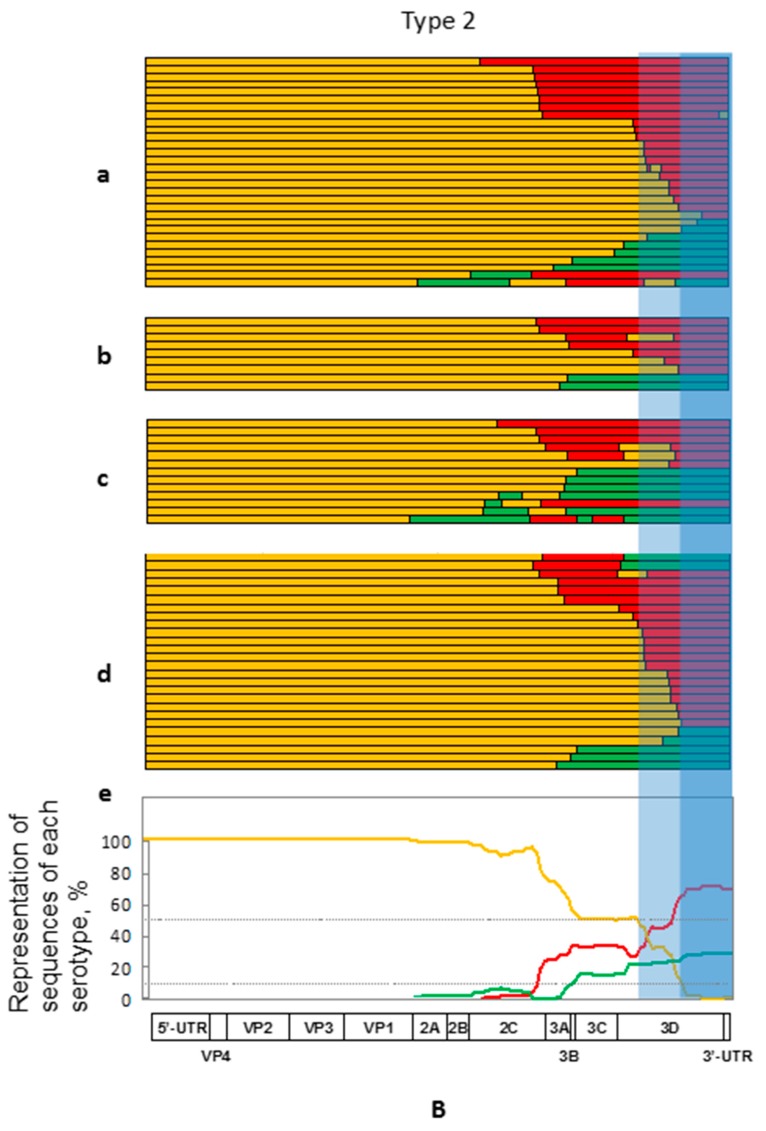

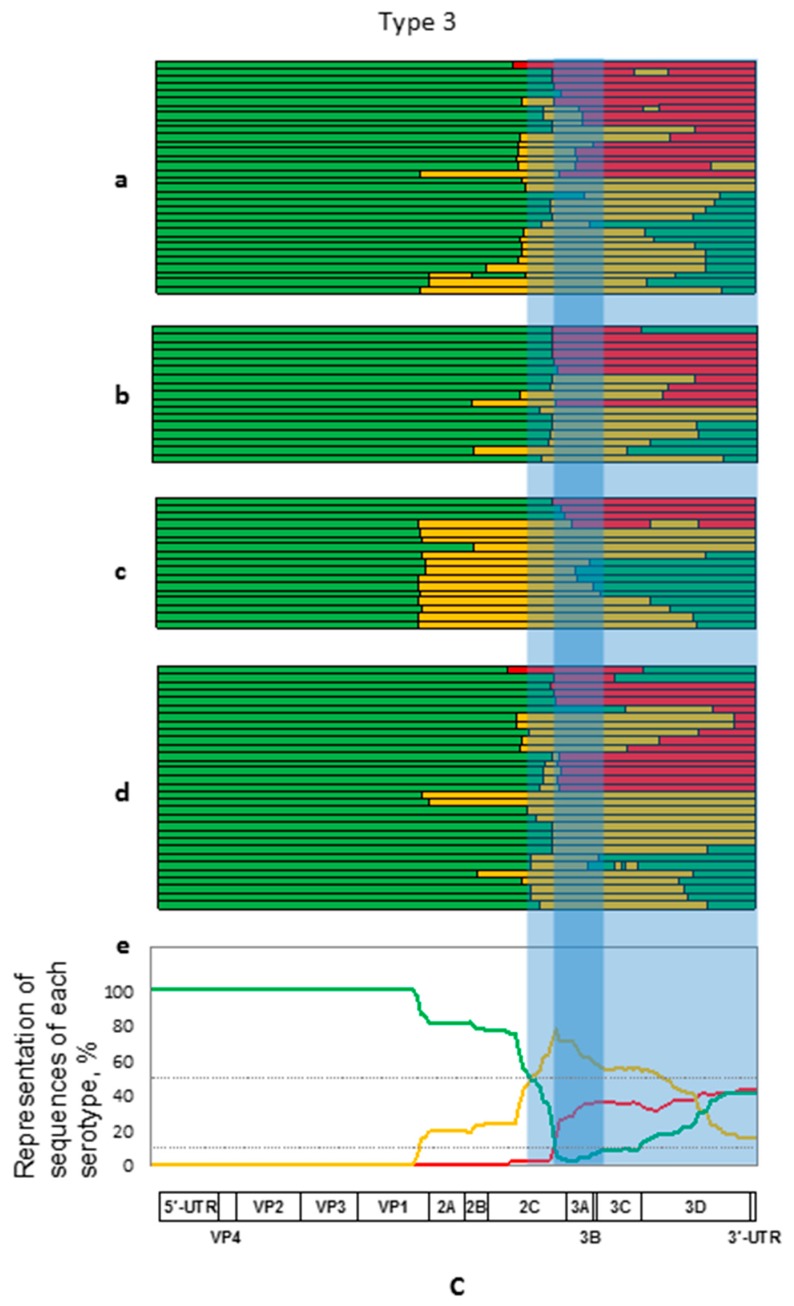
Schematic representation of the genome structures of recombinants of serotypes 1 (**A**), 2 (**B**), and 3 (**C**). Sequences derived from these serotypes are depicted in red, orange, and green respectively. In each panel, the genomes are shown that were sequenced fully (*a*) and partially (*b*) in this study, fully sequenced from Genbank (*c*), and partially sequenced previously (*d*). The occurrence of additional crossovers in the partially sequenced genomes could not be ruled out. The presence of foreign sequences in the genomes of recombinants of a given serotype is summarized in (*e*). The functional genetic map of the poliovirus genome is given at the bottom of each panel. Heavier and lighter shading refer to the proposed “weak” and “provisionally weak” genomic areas (see text). UTR, untranslated region.

The majority of recombinants of serotype 1 had the first crossover points close to the border between the regions encoding structural and nonstructural proteins, resulting in the replacement of significant portions of the genome by sequences derived from serotype 2 in most cases (Figure 1A). The size of serotype 2-derived fragments varied between ~600 to >3000 nt. All of these viruses had also at least one additional crossover, predominantly leading to the acquisition of the serotype 1-derived 3′-terminal parts of the genome, demonstrating preference for having homotypic genome ends.

In contrast, all but one recombinants of serotype 2 had heterotypic genome ends with a commensurable representation of type 1- and type 3-derived sequences (Figure 1B). The majority of recombinants had a single crossover located in many cases downstream of the central P2 genomic region. However, 10 out of 78 recombinants of this serotype appeared to have as many as 4–5 crossovers.

Recombinants of serotype 3 demonstrated a more variable pattern of the inheritance of heterotypic sequences (Figure 1C). A sizeable proportion of genomes acquired type 2 sequences around the structural/nonstructural border but in the majority of cases the crossovers were located more downstream within the P2 (i.e., 2A-C-encoding) region, resulting in the acquisition of either type 2- or type 1-specific sequences (the latter preferably in the case of relatively more distal crossovers). 3′-proximal parts of the gained serotype 3-specific sequences might or might not be replaced through additional recombination events by type 1 or type 2 sequences, giving rise to more or less comparable proportions of the genomes with either homotypic or heterotypic (mostly serotype 1-derived) 3′-terminal genome segments. Some recombinants of this serotype also had more than 2 crossover points.

### 3.3. Preferential Type-Specific Location of the Lost Genomic Regions

To ascertain the biological relevance of recombination events, it is important to analyze not only the acquired but also the lost genetic information. In the recombinants of serotype 1, the deleted type-specific sequences mapped predominantly to the region spanning between the capsid genes and internal parts of the *3D^pol^* gene, with an apparent trend to retain, in some cases, their own 3ABC and surrounding sequences, suggesting the possibility that there are two most likely regions undergoing removal located around the 2B/2C and 3C/3D borders respectively (Figure 1A). It should be admitted however that the number of recombinants of this serotype was too small to make firm conclusions. In serotype 2 recombinants, distal portions of the 3D*^pol^*-coding and 3′-untranslated region (UTR) sequences were regularly lost, in some cases together with more or less extended parts of the P3 region and sometimes also distal portions of the P2 region (Figure 1B). Serotype 3 recombinants demonstrated, as noted above, a less regular pattern of losing their own information, with the removal of (roughly) P2- or P3-related type-cognate sequences or both (Figure 1C). The absence of short type-specific sequences within the 3A-coding region was a common feature of all but a couple of members of this group. The common absence of this genome piece could be due to either its inherent properties or to the overlapping of the two just mentioned larger segments.

**Figure 2:**
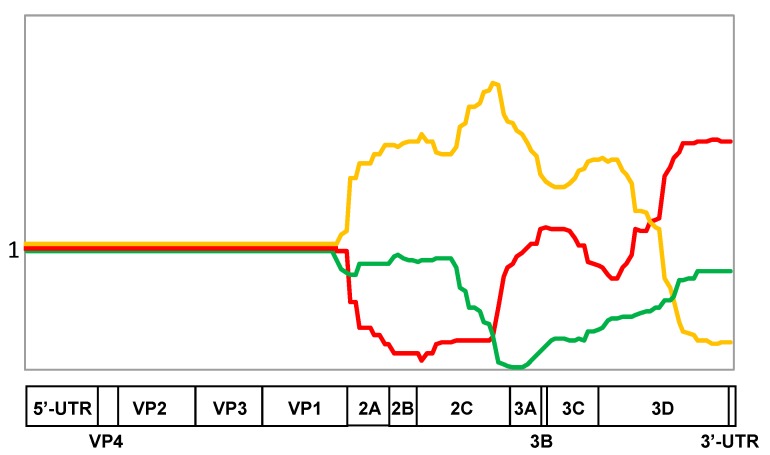
The occurrence of type-specific genomic segments in the normalized common set of recombinants of all three serotypes. The set of recombinants of each serotype is normalized to 1. Sequences derived from these serotypes are depicted in red, orange, and green respectively.

The above results indicated that the patterns of intertypic recombination of Sabin viruses are not haphazard but rather are markedly regular. Although some contribution of chance events, for example, bottlenecking effects during viral transmission, cannot be ignored, these patterns are likely determined by more profound circumstances. In principle, being serotype-specific, they could be due to two not mutually exclusive reasons: the lost segments might be either intrinsically weak (low-fit) or poorly compatible with other genome parts (epistasis). Some ground for choosing the more likely explanation can provide a comparison of data of Figure 1 with those of Figure 2, in which the abundance of different type-specific genomic segments in the normalized common set of recombinants of all three serotypes is presented.

Thus, the presence of homotypic 5′- and 3′-adjacent sequences in the type 1 recombinants (Figure 1A) could be hypothetically due to their preferred mutual compatibility (epistasis) but, as demonstrated in Figure 2, this 3′-terminal sequence was evidently good for all serotypes, suggesting that it may possess intrinsically higher fitness. Similarly, some other distinct portions of each serotype appeared to exhibit either relatively high (a significant part of the serotype type 2 sequences encoding nonstructural proteins, especially a 3′-proximal region of the 2C gene) or low preference (serotype 2 3′-terminus and serotype 3 sequence around the border between the P2 and P3 regions) irrespective of the nature of capsid proteins. Thus, the relative intrinsic weakness/strength of distinct genomic segments appeared to play a significant role in their loss/acquisition by recombination, though effects of epistatic interactions could not be discounted as well.

The notion of the existence of presumably low-fit serotype-specific segments of poliovirus genomes is also supported by the structures of recombinants between Sabin strains and non-polio enteroviruses. Thus, for example, such recombinants of type 1, which triggered polio outbreaks in the Dominican Republic and Philippines in 2000–2001, demonstrated a replacement of a significant portion of their P2 region [25,26]; the genomes of type 2 recombinants involved in several outbreaks were deprived of their homotypic 3′-ends [27,28]; and polio-nonpolio recombinants of type 3 circulating in Madagascar possess the genome structure similar to those of intertypic recombinants of this serotype described above [29].

### 3.4. Peculiarities of the Eliminated Genomic Regions

**Figure 3:**
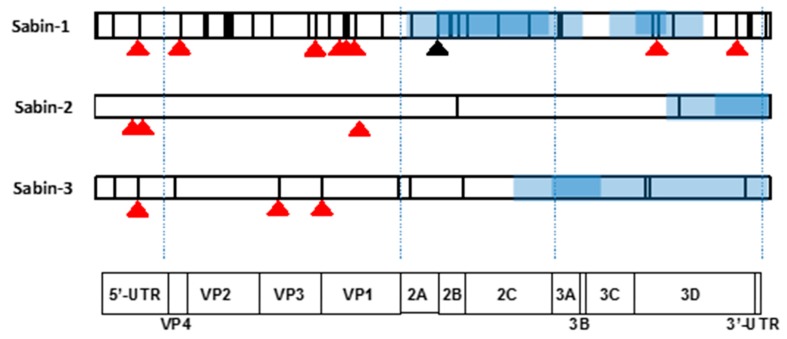
Location of known attenuating mutations (red triangles), an often-reverted serotype-1-specific mutation (black triangle) and mutations acquired upon the derivation of vaccine strains (vertical lines) in the genomes of Sabin viruses. Regions speculated to determine a decreased fitness are highlighted as blue rectangles. Their darker and lighter shading refer to the proposed “weak” and “provisionally weak” genomic areas (see Figure 1). The schematic representation of poliovirus genome and its functional elements are given at the bottom.

A plausible assumption is that the observed regularity of losing/gaining distinct genomic segments is linked to their fitness-modulating capacities. Are they somehow related to the type-specific attenuating mutations acquired upon derivation of the vaccine strains? To approach this point, we used an arbitrary classification of presumably low-fit regions based on the frequency of their removal upon intertypic recombination. Those that were lost in 90% or more recombinants of serotypes 2 and 3 and in at least 75% of serotype 1 were considered as “weak” (heavier shading in Figure 1 and Figure 3), whereas those that were absent less often but still in at least a half of the recombinants were regarded “provisionally weak”. A less stringent criterion for serotype 1 was due to a relatively small set of such recombinants. It goes without saying that the actual borders of low-fit regions may not exactly coincide with such arbitrary parameters, which merely point to their supposed approximate locations.

As shown in Figure 3, all (for serotypes 2 and 3) or the majority (for serotype 1) of known attenuating (and temperature-sensitive (*ts*) phenotype-modulating) mutations are located upstream of the recombination-affected parts of the genomes situated, as demonstrated above, in our set of viruses downstream of the VP1/2A border. Moreover only a single known Sabin 1-specific attenuating mutation (position 6203 in the 3D-encoding gene [30,31] mapped to a “weak” region. Importantly, a significant proportion of the known attenuating mutations of all serotypes had been deattenuated independently of the recombination events (Table 2). These facts suggest that both recombination and single-based reversions of the known attenuating mutations (or compensating alterations) could lead to restoration of neurovirulence.

The hypothetical possibility also exists that recombination could be linked to the removal of other mutations selected upon derivation of vaccines. In the case of serotype 1, the mutations distinguishing Sabin-1 from its wild-type parent (strain Mahoney) are scattered throughout the entire genome (Figure 3), and therefore this hypothesis could not be adequately assessed. Nevertheless, it may be noted that in addition to the attenuating mutation mentioned above, another Sabin-1-specific mutation (Thr instead of wild type Ser at position 134 of the 2A protein) with the propensity to revert to the wild type residue [33,34] mapped to one of the “weak” regions frequently lost by recombination. The contribution to the viral biology, if any, of other Sabin-1-specific mutations located in the "provisionally weak” parts is unknown. It should be again admitted that the set of the serotype 1 intertypic recombinants with known sequenced genomes is not sufficiently large to make definite conclusions. However, the hypothesis about the dependence of the recombination crossovers on the mutations acquired upon derivation of the Sabin vaccine was hardly supported by recombinants of serotypes 2 and 3, in which only few (one and three, respectively) vaccine-specific mutations without any reported phenotypic effects were located within “provisionally weak” segments (Figure 3).

**Figure 4:**
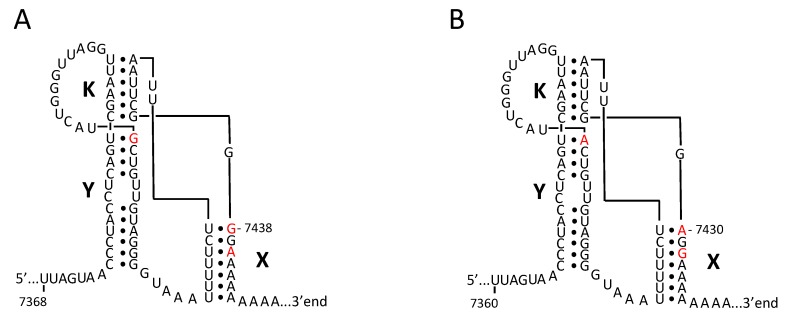
Schematic representation of the possible 3′-terminal tertiary structures of the RNAs of Sabin-2 (**A**) and Sabin-3 (**B**) polioviruses. It shows the helical elements of hairpins X and Y as well as the tertiary kissing interaction between these hairpins (K). The nucleotides distinguishing these two 3′UTRs are highlighted in red. Of note, these distinctive nucleotides in Sabin 1 RNA are the same as in Sabin 3. Modified from [35].

Thus, it is reasonable to look at the intrinsic (not altered upon the derivation of vaccine strains) peculiarities of the “weak” genomic segments. The regular absence of the type 2-specific 3′UTR in the investigated recombinants could be due to certain peculiarities of the structure of this replicative *cis*-element (*ori*R), which in poliovirus RNA is composed of two hairpins linked to each other by a tertiary (“kissing”) interaction [35]. Although the spatial organization of the 3′UTRs of all Sabin viruses appears to be highly conserved, serotype 2 possesses two potentially important distinctions: stems of its hairpins X and Y end with U-G pairs instead of U-A pairs in two other serotypes (Figure 4). These distinctions are expected to partially destabilize the tertiary structure of *ori*R. Whether one or both of these features could be linked to fitness loss remains unknown. It may also be noted that the distal part of the 3D protein invariably lost in the serotype 2 recombinants differs from its counterparts in types 1 and 3 at only one position, amino acids 362 and 425, respectively. The loss of this segment could, or could not, be simply due to a kind of “passenger effect” because of its proximity to the 3′UTR.

The presence of Arg at position 15 of 3A protein of Sabin-3 could also be related to the removal of this region from nearly all type 3 recombinants. Indeed, none of the wild polioviruses deposited in the GenBank (except the Sabin-3 parent Leon/37 and its descendants) contain Arg_15_ in 3A. On the other hand, as noted above, it cannot be ruled out that a nearly universal loss of the relevant short fragment resulted merely from the partial overlap of two low-fitness regions located in P2 and P3, respectively.

Thus, the above results are compatible with the hypothesis that the fitness-related features of the genome segments that are acquired/lost upon intertypic recombination between Sabin polioviruses might be determined by not only vaccine-specific mutations but could also be inherited from their wild type progenitors.

### 3.5. Characterization of the Crossover Sites

To better evaluate the choice of the observed crossover sites, it is appropriate to briefly remind what is known about the mechanisms of RNA recombination [36]. The widely accepted model of intermolecular recombination between RNA viruses is replicative template switching (”copy-choice”), whereby an incomplete part of the newly-synthesized strand is displaced from the original template and transferred to another one to serve as a primer for the continuation and completion of the genome synthesis [37,38]. An important feature of this model is the requirement for homology (or close similarity) of the genomic regions of the recombining partners around the to-be-formed crossovers. This homology is needed to ensure proper landing of the 3′-terminal portion of the departing incomplete RNA segment onto the new template. A variant of the replicative RNA recombination was also proposed, which does not require such homology [39]. In this model, drawing together the original and the second template strands is ensured by their binding to the neighboring sites of a third (unrelated) “guide” RNA strand. Consequently, the viral RNA-dependent RNA polymerase is given a chance to jump from the donor template to the recipient one without dissociation. However, the existence of such “guides” with neighboring sequences complementary to the relevant parts of recombining partners is hardly expected to be a frequent phenomenon.

Another, nonreplicative, mechanism of RNA recombination implies end-to-end joining of independently synthesized RNA fragments without participation of viral RNA-dependent RNA polymerase (RdRP) and does not require any similarity between the partners involved [40,41]. Although all three above mechanisms are likely to play roles in shaping evolution of RNA viruses, their involvement or non-involvement in particular natural recombination events is usually hypothetical, at best. Some preliminary information in this regard could be obtained by analyzing the structures of crossover sites. In any case, the knowledge of the nucleotide sequences around the crossovers is important for understanding the regularities of their location.

**Figure 5:**
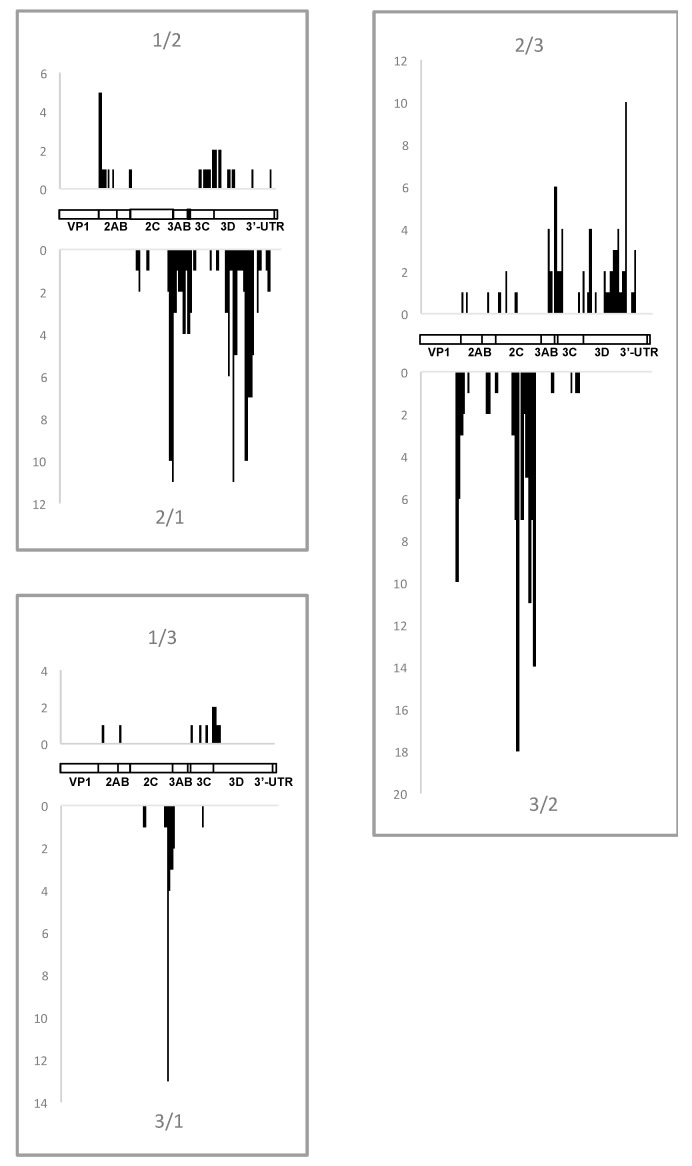
The location of all intertypic crossover sites in the analyzed recombinants. The number of respective crossovers is given on the vertical axes. Schematic genome maps are also presented. In this and the following Figure the data from [12] are also included.

Although the choice of the replaced segments appears to be related to fitness, there should be mechanistic circumstances to allow recombination events to occur at certain distinct loci. The available large collection of recombinants originating from known parents may be useful for a search for such mechanistic preferences/limitations, if they exist at all. In our set of recombinants, the crossovers were distributed throughout the entire region encoding nonstructural proteins, though clearly non-randomly with relatively “hot” and “cold” areas (Figure 5). Remarkably, distribution of crossovers was type-specific, and, importantly, not similar in the reciprocal pairs of recombinants, i.e., differed between type X/type Y and type Y/type X recombinants, suggesting that the match of nucleotide stretches (if really important) was not the only factor in the choice of crossover sites.

**Figure 6:**
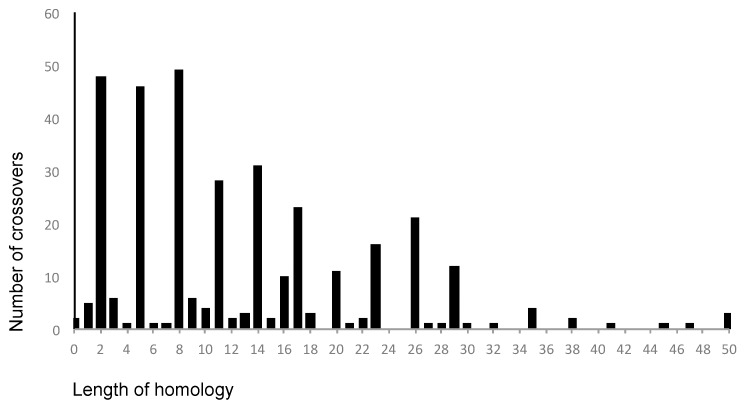
The dependence of the frequency of crossovers on the length of homologous sequences in the relevant regions of the recombining partners. Peaks corresponding to homology lengths of 2, 5, 8, etc. are caused by the fact that the most often difference between sequences of 3 serotypes concerns the third positions of the codons.

The above pattern of crossover distribution was generated manually using 50 nt-long windows by comparing aligned type-specific sequences. To assess the real level of homology around crossover points in the recombining partners, we looked at the sequences of relevant genomes. It turned out that in the overwhelming majority of cases (81%), this homology indeed spanned for at least 4 nt and in 68% cases it was 7 nt-long or longer (Figure 6). This may be expected because of a significant similarity between genomic sequences of non-structural regions of all three serotypes, and therefore cannot be used as a strong argument to reveal the underlying mechanism of recombination (though homology is an obvious requirement for the replicative mode). However, a substantial proportion (19%) of recombining partners had only 3 or less (even none) common nt around the crossovers, which is hardly compatible with the classical replicative models of RNA recombination [37,38], suggesting possible involvement of some additional mechanisms, for example, those mentioned above.

### 3.6. General Remarks: Possible Biological Relevance

The data reported here revealed previously unknown serotype-specific peculiarities of distinct genomic segments of poliovirus vaccine strains, which are likely related to their effects on viral fitness. Although polio eradication campaign launched by the WHO in 1988 (World Health Assembly, 1988) met with a marked success, with only a handful of poliomyelitis cases caused by wild poliovirus reported globally in 2017 [42], the need for vaccination against the disease will remain for the foreseeable future [43,44]. The existing excellent vaccines, the Sabin OPV and Salk inactivated polio vaccine (IPV), both have well known drawbacks. OPV strains can evolve into more pathogenic variants and inflict paralytic disease [6,7,8,14], while IPV does not induce robust intestinal immunity and is unable to interrupt viral transmission [45]. Therefore, different attempts to develop better vaccines, including live ones, are in progress [46,47,48,49,50,51]. A critically important characteristic of new attenuated strains should be their genetic stability.

Unfortunately, phenotypic differences between Sabin viruses and their intertypic recombinants have not been systematically investigated. In a small-scale experiment, Savolainen-Kopra et al. [52] have demonstrated that a Sabin-2/Sabin-1 recombinant outcompeted its serotype-2 parent in tissue culture experiments, but whether such preference is a general rule is unknown. Moreover, in vitro experiments are not necessarily sufficient to give the proper answer about the properties and behavior of viruses in human populations. Even though there is no conclusive evidence linking recombination with increased virulence, its involvement is supported by the observation that the majority of VDPV strains are recombinants. Therefore type-specific recombination patterns described here may be relevant.

To decrease the ability of vaccine strains to recombine it was suggested to introduce certain mutations into the viral RNA-dependent RNA polymerase [46] and to use a common (e.g., Sabin-3-derived) sequence downstream from the capsid region as a backbone for all serotypes [49]. The results reported here suggest an additional approach: generation of strains with the noncapsid genomic regions not only identical in all three serotypes but also possessing the highest possible fitness. These regions could be composed of “strong” segments from all three serotypes. Engineered attenuating mutations in the 5′UTR and P1 regions would probably be sufficient to achieve the target level of attenuation. Such vaccine strains can be expected to be more genetically stable and less prone to recombination not only with each other (due to the identity of a major part of the genome) but, possibly, with other enteroviruses as well (due to their improved fitness). However, the effect of such arrangement of genomic segments on viral neurovirulence must be studied. On the other hand, one may also ask whether engineered strains containing a combination of only “weak” non-structural genomic region are attenuated to such a degree that their residual neurovirulence is completely lost. Tentative answer to these important questions could be obtained in experiments in poliovirus-susceptible animals.

A likely pattern emerging from the results presented here is that naturally circulating (“wild”) RNA viruses may contain lineage-specific more or less extended genomic segments with a relatively low fitness. Indeed, our observations suggest that Sabin viruses (at least of serotype 2 or 3) might have inherited from their wt parents certain “weak” RNA sequences not related to the attenuating (and other) mutations acquired during their selection by A. Sabin. Such low-fitness segments could well arise (and probably indeed are not infrequently arising) upon error-prone RNA replication caused by low fidelity of the RdRP (reviewed in [53,54]) and regular intermolecular recombination [55,56]. Notwithstanding their selective disadvantage, such viruses could be founders of novel lineages due to various bottlenecking events during their replication and transmission. Decreased fitness of some of such segments could be due to multiple mutations, phenotypic effects of which could be interdependent (epistasis). In certain cases, their rehabilitation could not be readily achieved by isolated reversions or compensatory mutations. The most feasible way to solve this problem could be recombination with an appropriate partner devoid of this particular source of deficiency. Obviously, there should be only rare opportunities to find such a partner during natural circulation. In fact, the above hypothesis could have been formulated just because of the mega-experiment of co-infecting billions of susceptible hosts simultaneously with three distinct lineages of a virus. It may be suggested that such low-fitness segments are a relatively common feature of RNA viruses, confirming that recombination may serve a significant role in evolution of such viruses.

## 4. Concluding Remarks

The analysis of the pattern of inheritance of distinct genomic segments upon intertypic recombination between the Sabin strains of poliovirus allowed us to draw several conclusions and hypotheses. It suggests that Sabin strains contain, in addition to known attenuating mutations, distinct serotype-specific genomic segments with low-fitness. The genetic determinants responsible for their low fitness could be derived not only during the laboratory attenuation of these strains but also could be inherited directly from their wild-type parents. The presence of these segments underlies the propensity of these viruses to recombine with each other and with other enteroviruses as well as makes a significant contribution to the choice of crossovers sites. Thus, our results clearly demonstrate a role for RNA recombination as an efficient tool of adaptive viral evolution. Also, the knowledge of localization of low-fitness segments in Sabin viruses opens additional possibilities for the design of more genetically stable and/or more attenuated vaccine strains.

Our results also provide new insights into the problem of fitness, genetic stability and evolvability of wild polioviruses and, by generalization, of other RNA viruses. We propose that the genomes of such viruses may also harbor hidden low-fitness segments, which cannot be readily rehabilitated and can (nearly) exclusively be removed by recombination.

Finally, the absence of sufficiently long homologous sequences around crossover sites in the relatively large pairs of recombining partners may be considered as an indirect evidence for possible involvement, in certain cases, of recombination mechanisms other than the classical copy-choice replicative mode. However, more direct data to support this notion are needed.

## Figures and Tables

**Table 1:** Overview of the set of analyzed intertypic recombinants.

	Our Study		Published Data		Total
**Methods of Analysis**	**Full Genome Sequencing**	**Partial Genome Sequencing**	**Full Genome Sequencing**	**Partial Genome Sequencing**	
Number of isolates	70	28	33	58	189
Number (%) of type 1, type 2, and type 3 isolates	8 (11),	2 (7),	3 (9),	1 (2),	14 (8),
	30 (43),	9 (32),	13 (39),	26 (45),	78 (41),
	32 (46)	17 (61)	17 (52)	31 (53)	97 (51)
Number (%) of VDPV	5 (7)	?	15 (45)	?	?
Number (%) of Sabin-like strains	65 (93)	?	18 (55)	?	?
Number (%) of AFP cases	40 (57)	20 (71)	24 (73)	20 (34)	104 (55)
Number (%) of isolates from patients with other diagnoses	6 (9)	4 (14)	1 (3)	-	11 (6)
Number (%) of isolates from healthy persons	12 (17)	1 (4)	7 (21)	16 (28)	36 (19)
Number (%) of isolates from sewage	9 (13)	3 (11)	1 (3)	11 (19)	24 (13)
Number (%) of strains of unknown origin	3 (4)	-	-	11 (19)	14 (7)

VDPV, vaccine-derived poliovirus; AFP, acute flaccid paralysis.

**Table 2:** Loss of attenuating and/or temperature-sensitive (*ts*) mutations in the set of recombinants with the fully sequenced genomes.

Serotype	Location of Mutation	Attenuating Mutations Lost, %	
		In the Retained Part of the Genome	In the Removed Part of the Genome
1	480	67	-
	525	11	-
	935	22	-
	2438	33	-
	2741	22	-
	2795	67	-
	2879	0	-
	6203	9	82
	7071	8	25
2	398	49	-
	481	100	-
	2908	5	-
	2909	85	-
3	472	98	-
	2034	20	-
	2493 ^a^	100	-

^a^ 2493-U is present in some vaccines [32], therefore this mutation does not necessarily represent reversion during several virus transmission.

## Supplementary Materials

The following are available online at www.mdpi.com/1999-4915/9/11/353/s1, Table S1: Recombinants whose full genome sequences were determined in this study, Table S2: Recombinants whose partial genome sequences were determined in this study, Table S3: Recombinants whose full genome sequences were reported previously, Table S4: Recombinants whose partial genome sequences were reported previously.
