# Supplementary material for: Pressure for Pattern-Specific Intertypic Recombination between Sabin Polioviruses: Evolutionary Implications
Source: Viruses. 2017 Nov 22;9(11):353. doi: 10.3390/v9110353 (PMC5707560; doi:10.3390/v9110353)
Supplement: Supplementary file 1 [file viruses-09-00353-s001.zip › Table S1.docx]

# Table S1. Recombinants whose full genome sequences were determined in this study

| **#** | **Lab. # of isolate** | **Source** | **% nucleotide substitutions in VP1** | **VDPV or Sabin-like** | **Recombinant structure** |
| --- | --- | --- | --- | --- | --- |
| **Serotype 1** | | | | | |
| 1 | 18339 | AFP | 0.55 | Sabin-like | S1-S2-S1 |
| 2 | 8757 | AFP | 0.88 | Sabin-like | S1-S2-S1 |
| 3^a^ | 29690_c1 |  | 0.44 | Sabin-like | S1-S2-S1 |
| 4^a^ | 29690_c6 | healthy | 0.44 | Sabin-like | S1-S2-S1 |
| 5^a^ | 29690_c9 |  | 0.44 | Sabin-like | S1-S2-S1 |
| 6 | 28698 | sewage | 0.22 | Sabin-like | S1-S2-S1 |
| 7 | 1PMR046018 | sewage | 0.33 | Sabin-like | S1-S2-S1 |
| 8 | 13688 | unknown | 0 | Sabin-like | S1-S3-S2-S1-S2-S3 |
| **Serotype 2** | | | | | |
| 9 | 15767_T2_c0 | AFP | 0.11 | Sabin-like | S2-S3 |
| 10 | 17719_T2 | AFP | 0.11 | Sabin-like | S2-S3 |
| 11 | 18058 | AFP | 0.11 | Sabin-like | S2-S1 |
| 12 | 19062 | AFP | 0.11 | Sabin-like | S2-S1 |
| 13 | 21348_T2 | AFP | 0.11 | Sabin-like | S2-S3 |
| 14 | IS_061 | AFP | 0.11 | Sabin-like | S2-S1 |
| 15 | 12604 | AFP | 0.22 | Sabin-like | S2-S3 |
| 16 | 13770 | AFP | 0.22 | Sabin-like | S2-S1 |
| 17 | 15763_T2 | AFP | 0.22 | Sabin-like | S2-S1 |
| 18 | 16849 | AFP | 0.22 | Sabin-like | S2-S1 |
| 19 | 17629 | AFP | 0.22 | Sabin-like | S2-S3 |
| 20 | IS_001 | AFP | 0.22 | Sabin-like | S2-S3 |
| 21 | 9112 | AFP | 0.33 | Sabin-like | S2-S1 |
| 22 | 9632_T2 | AFP | 0.33 | Sabin-like | S2-S1 |
| 23 | 15767_T2_c1 | AFP | 0.44 | Sabin-like | S2-S1 |
| 24 | 14337 | AFP | 0.55 | Sabin-like | S2-S1-S2 |
| 25 | 18580 | AFP | 0.55 | Sabin-like | S2-S1 |
| 26 | 19890 | AFP | 2.77 | VDPV | S2-S3-S2-S1-S2-S3 |
| 27 | 12209 | viral infection | 0.66 | VDPV | S2-S1 |
| 28 | 14304 | sewage | 0.11 | Sabin-like | S2-S1 |
| 29 | 6M_V | sewage | 0.11 | Sabin-like | S2-S3 |
| 30^a^ | 29425_c0 | healthy | 0 | Sabin-like | S2-S1 |
| 31^a^ | 29425_c11 |  | 0 | Sabin-like | S2-S1 |
| 32^a^ | 29683_c0 | healthy | 0 | Sabin-like | S2-S1 |
| 33^a^ | 29683_c24 |  | 0 | Sabin-like | S2-S1-S2-S1 |
| 34 | 14732 | healthy | 0.11 | Sabin-like | S2-S3-S1 |
| 35 | 31947 | healthy | 0.44 | Sabin-like | S2-S1 |
| 36 | 10630 | healthy | 0.66 | VDPV | S2-S1 |
| 37 | 10 | unknown | 0.22 | Sabin-like | S2-S1 |
| 38 | 7 | unknown | 3.65 | VDPV | S2-S1 |
| **Serotype 3** | | | | | |
| 39 | 14385 | AFP | 0.11 | Sabin-like | S3-S1 |
| 40 | 15770 | AFP | 0.11 | Sabin-like | S3-S1-S2-S1 |
| 41 | 19017 | AFP | 0.11 | Sabin-like | S3-S2-S3 |
| 42 | 9633_T3 | AFP | 0.22 | Sabin-like | S3-S1 |
| 43 | 14770 | AFP | 0.22 | Sabin-like | S3-S2-S1 |
| 44 | 14829 | AFP | 0.22 | Sabin-like | S3-S2-S1 |
| 45 | 14984 | AFP | 0.22 | Sabin-like | S3-S2-S1-S2-S1 |
| 46 | 15767_T3 | AFP | 0.22 | Sabin-like | S3-S2-S3 |
| 47 | 17719_T3 | AFP | 0.22 | Sabin-like | S3-S2-S3 |
| 48 | 17723 | AFP | 0.22 | Sabin-like | S3-S2-S3 |
| 49 | 21348_T3 | AFP | 0.22 | Sabin-like | S3-S2-S3 |
| 50 | 10073 | AFP | 0.33 | Sabin-like | S3-S2 |
| 51 | 16938 | AFP | 0.33 | Sabin-like | S3-S2-S3 |
| 52 | 21390 | AFP | 0.33 | Sabin-like | S3-S2-S1 |
| 53^a^ | 15762 | AFP | 0.44 | Sabin-like | S3-S2-S1 |
| 54^a^ | 15763_T3 |  | 0.44 | Sabin-like | S3-S2-S1 |
| 55 | 18963 | AFP | 0.44 | Sabin-like | S3-S2-S3-S2-S3 |
| 56 | 10664 | AFP | 0.56 | Sabin-like | S3-S2-S1 |
| 57 | 11948 | AFP | 0.67 | Sabin-like | S3-S2-S3 |
| 58 | 16204 | AFP | 0.67 | Sabin-like | S3-S2-S3 |
| 59^a^ | 32335_c0 |  | 0.44 | Sabin-like | S3-S2-S3 |
| 60^a^ | 32335_c5 | acute | 0.44 | Sabin-like | S3-S2-S3 |
| 61^a^ | 32335_c8 | enterocolitis | 0.44 | Sabin-like | S3-S2-S1 |
| 62^a^ | 32335_c17 |  | 0.44 | Sabin-like | S3-S2-S1-S2 |
| 63^a^ | 32335_c19 |  | 0.44 | Sabin-like | S3-S2-S1 |
| 64 | 29813 | healthy | 0.56 | Sabin-like | S3-S2-S1 |
| 65 | 11264 | healthy | 1.89 | VDPV | S3-S2-S3 |
| 66 | 2K_P9 | sewage | 0.11 | Sabin-like | S3-S1 |
| 67 | 23_S2_P_6-4 | sewage | 0.33 | Sabin-like | S3-S2 |
| 68 | 36_S2_P_7-3 | sewage | 0.33 | Sabin-like | S3-S2-S3 |
| 69 | S20PMR46017 | sewage | 0.33 | Sabin-like | S3-S1 |
| 70 | S16PMR46022 | sewage | 0.56 | Sabin-like | S3-S2-S3 |

^a^ Different recombinants were isolated from the same persons.
