# Supplementary material for: Pressure for Pattern-Specific Intertypic Recombination between Sabin Polioviruses: Evolutionary Implications
Source: Viruses. 2017 Nov 22;9(11):353. doi: 10.3390/v9110353 (PMC5707560; doi:10.3390/v9110353)
Supplement: Supplementary file 1 [file viruses-09-00353-s001.zip › Table S2.docx]

# Table S2. Recombinants whose partial genome sequences were determined in this study

| # | **Lab. # of isolate** | **Source** | **Known % nucleotide substitutions in VP1** | **Recombinant structure** |
| --- | --- | --- | --- | --- |
| **Serotype 1** | | | | |
| 1 | 8472 | sewage | 0.33% | S1-S2-S1 |
| 2 | 29626 | sewage | - | S1-S2-S1-S2-S1 |
| **Serotype 2** | | | | |
| 3 | 9374 | AFP | - | S2-S1 |
| 4 | 12889 | AFP | - | S2-S1 |
| 5 | 29314 | AFP | - | S2-S1 |
| 6 | Pe-o | AFP | - | S2-S1 |
| 7 | Pi-o | AFP | - | S2-S1 |
| 8 | Po-o | AFP | - | S2-S1 |
| 9^a^ | 6074(1) | AFP | - | S2-S3 |
| 10^a^ | 6074(2) |  | - | S2-S3 |
| 11 | 950 | encephalitis | - | S2-S1-S2-S1 |
| **Serotype 3** | | | | |
| 12 | 7532 | AFP | 0.67 | S3-S1-S3 |
| 13 | 9220 | AFP | - | S3-S2-S3 |
| 14 | 9586 | AFP | - | S3-S2 |
| 15 | 10279 | AFP | - | S3-S1 |
| 16 | 10581 | AFP | - | S3-S2-S3 |
| 17 | 10618 | AFP | - | S3-S2 |
| 18 | 10859 | AFP | - | S3-S2-S3 |
| 19 | 11624 | AFP | - | S3-S1 |
| 20 | 11883 | AFP | - | S3-S2-S1 |
| 21 | 12605 | AFP | - | S3-S2-S3 |
| 22 | 13222 | AFP | - | S3-S1 |
| 23 | 14765 | AFP | - | S3-S2-S1 |
| 24 | 6061 | congenital encephalopathy | 0.11 | S3-S2-S3 |
| 25 | 8148 | somatic disease | - | S3-S1 |
| 26 | 9162 | polio-like disease | - | S3-S1 |
| 27 | 29451 | healthy | 0.33 | S3-S2-S1 |
| 28 | 28086 | sewage | - | S3-S2-S1 |

^a^ Different recombinants were isolated from the same patient.
