# Supplementary material for: Pressure for Pattern-Specific Intertypic Recombination between Sabin Polioviruses: Evolutionary Implications
Source: Viruses. 2017 Nov 22;9(11):353. doi: 10.3390/v9110353 (PMC5707560; doi:10.3390/v9110353)
Supplement: Supplementary file 1 [file viruses-09-00353-s001.zip › Table S3.docx]

# Table S3. Recombinants whose full genome sequences were reported previously

| # | **Genbank # of isolate** | **Source** | **% nucleotide substitutions in VP1** | **VDPV or Sabin-like** | **Recombinant structure** | **Reference** |
| --- | --- | --- | --- | --- | --- | --- |
| Serotype 1 | | | | | | |
| 1 | AF462419 | AFP | 0.44 | Sabin-like | S1-S2-S1 | [[1](#_ENREF_1)] |
| 2 | FJ859061 | AFP | 1.66 | VDPV | S1-S2-S1-S3 | [[2](#_ENREF_2)] |
| 3 | AF462418 | AFP | 2.65 | VDPV | S1-S2-S1 | [[1](#_ENREF_1)] |
| Serotype 2 | | | | | | |
| 4 | JX275266 | AFP | 0.66 | VDPV | S2-S1 | [[3](#_ENREF_3)] |
| 5 | JX275352 | AFP | 0.66 | VDPV | S2-S1 | [[3](#_ENREF_3)] |
| 6 | JX274981 | AFP | 0.66 | VDPV | S2-S3 | [[3](#_ENREF_3)] |
| 7 | EU566950 | AFP | 0.89 | VDPV | S2-S1-S2-S1 | [[4](#_ENREF_4)] |
| 8 | HM107832 | AFP | 1 | VDPV | S2-S3-S2-S3 | [[5](#_ENREF_5)] |
| 9 | JX274987 | AFP | 1 | VDPV | S2-S1 | [[3](#_ENREF_3)] |
| 10 | AY948201 | AFP | 1.11 | VDPV | S2-S3-S1-S3-S1-S3 | [[5](#_ENREF_5)] |
| 11 | HM107834 | AFP | 1.22 | VDPV | S2-S3 | [[5](#_ENREF_5)] |
| 12 | HM107833 | AFP | 1.33 | VDPV | S2-S3 | [[5](#_ENREF_5)] |
| 13 | FJ517649 | AFP | 1.44 | VDPV | S2-S3-S2-S1 | [[6](#_ENREF_6)] |
| 14 | AY278552 | healthy | 0.22 | Sabin-like | S2-S1-S2-S1 | [[7](#_ENREF_7)] |
| 15 | AY278550 | healthy | 0.78 | VDPV | S2-S1 | [[7](#_ENREF_7)] |
| 16 | KJ419277 | healthy | 1.11 | VDPV | S2-S3-S2-S3 | [[8](#_ENREF_8)] |
| Serotype 3 | | | | | | |
| 17 | FJ859186 | AFP | 0.11 | Sabin-like | S3-S2-S3 | [[9](#_ENREF_9)] |
| 18 | FJ859189 | AFP | 0.22 | Sabin-like | S3-S2-S1-S2-S1 | [[9](#_ENREF_9)] |
| 19 | FJ859188 | AFP | 0.22 | Sabin-like | S3-S2-S3 | [[9](#_ENREF_9)] |
| 20 | FJ859192 | AFP | 0.22 | Sabin-like | S3-S2 | [[9](#_ENREF_9)] |
| 21 | FJ859183 | AFP | 0.33 | Sabin-like | S3-S2-S3 | [[9](#_ENREF_9)] |
| 22 | FJ859191 | AFP | 0.33 | Sabin-like | S3-S2-S3 | [[9](#_ENREF_9)] |
| 23 | FJ859187 | AFP | 0.56 | Sabin-like | S3-S2-S3 | [[9](#_ENREF_9)] |
| 24 | FJ859184 | AFP | 0.56 | Sabin-like | S3-S2-S3 | [[9](#_ENREF_9)] |
| 25 | FJ859185 | AFP | 0.67 | Sabin-like | S3-S2-S3 | [[9](#_ENREF_9)] |
| 26 | GU180608 | AFP | 0.78 | Sabin-like | S3-S1 | [[10](#_ENREF_10)] |
| 27 | EU684057 | AFP | 2.11 | VDPV | S3-S1 | [[11](#_ENREF_11)] |
| 28 | EF456707 | neutropenia and sudden fever | 0.78 | Sabin-like | S3-S2 | [[12](#_ENREF_12)] |
| 29 | FJ460226 | healthy | 0.33 | Sabin-like | S3-S1 | [[13](#_ENREF_13)] |
| 30 | FJ859190 | healthy | 0.33 | Sabin-like | S3-S2-S3 | [[9](#_ENREF_9)] |
| 31 | FJ460227 | healthy | 0.67 | Sabin-like | S3-S2 | [[13](#_ENREF_13)] |
| 32 | AF541919 | healthy | 0.67 | Sabin-like | S3-S2-S3 | [[14](#_ENREF_14)] |
| 33 | GU256222 | sewage | 0.33 | Sabin-like | S3-S2-S3 | [[15](#_ENREF_15)] |

# References

1. Cherkasova, E. A.; Korotkova, E. A.; Yakovenko, M. L.; Ivanova, O. E.; Eremeeva, T. P.; Chumakov, K. M.; Agol, V. I., Long-term circulation of vaccine-derived poliovirus that causes paralytic disease. *Journal of Virology* **2002,** 76, 6791-6799.

2. Yan, D.; Li, L.; Zhu, S.; Zhang, Y.; An, J.; Wang, D.; Wen, N.; Jorba, J.; Liu, W.; Zhong, G.; Huang, L.; Kew, O.; Liang, X.; Xu, W., Emergence and localized circulation of a vaccine-derived poliovirus in an isolated mountain community in Guangxi, China. *Journal of Clinical Microbiology* **2010,** 48, (9), 3274-80.

3. Burns, C. C.; Shaw, J.; Jorba, J.; Bukbuk, D.; Adu, F.; Gumede, N.; Pate, M. A.; Abanida, E. A.; Gasasira, A.; Iber, J.; Chen, Q.; Vincent, A.; Chenoweth, P.; Henderson, E.; Wannemuehler, K.; Naeem, A.; Umami, R. N.; Nishimura, Y.; Shimizu, H.; Baba, M.; Adeniji, A.; Williams, A. J.; Kilpatrick, D. R.; Oberste, M. S.; Wassilak, S. G.; Tomori, O.; Pallansch, M. A.; Kew, O., Multiple independent emergences of type 2 vaccine-derived polioviruses during a large outbreak in northern Nigeria. *J Virol* **2013,** 87, (9), 4907-22.

4. Avellon, A.; Cabrerizo, M.; de Miguel, T.; Perez-Brena, P.; Tenorio, A.; Perez, J. L.; de Aragon, M. V.; Trallero, G., Paralysis case and contact spread of recombinant vaccine-derived poliovirus, Spain. *Emerg Infect Dis* **2008,** 14, (11), 1807-9.

5. Zhang, Y.; Yan, D.; Zhu, S.; Wen, N.; Li, L.; Wang, H.; Liu, J.; Ye, X.; Ding, Z.; Wang, D.; Zhu, H.; Chen, L.; Hou, X.; An, H.; Liang, X.; Luo, H.; Kew, O.; Xu, W., Type 2 vaccine-derived poliovirus from patients with acute flaccid paralysis in China: current immunization strategy effectively prevented its sustained transmission. *The Journal of infectious diseases* **2010,** 202, (12), 1780-8.

6. Yakovenko, M. L.; Korotkova, E. A.; Ivanova, O. E.; Eremeeva, T. P.; Samoilovich, E.; Uhova, I.; Gavrilin, G. V.; Agol, V. I., Evolution of the Sabin vaccine into pathogenic derivatives without appreciable changes in antigenic properties: need for improvement of current poliovirus surveillance. *Journal of Virology* **2009,** 83, (7), 3402-6.

7. Korotkova, E. A.; Park, R.; Cherkasova, E. A.; Lipskaya, G. Y.; Chumakov, K. M.; Feldman, E.; Kew, O. M.; Agol, V. I., Retrospective analysis of a local cessation of vaccination against poliomyelitis: a possible scenario for the future. *Journal of Virology* **2003,** 77, 12460-12465.

8. Yan, D.; Zhang, Y.; Zhu, S.; Chen, N.; Li, X.; Wang, D.; Ma, X.; Zhu, H.; Tong, W.; Xu, W., Limited and localized outbreak of newly emergent type 2 vaccine-derived poliovirus in Sichuan, China. *Clinical and vaccine immunology : CVI* **2014,** 21, (7), 1012-8.

9. Zhang, Y.; Zhu, S.; Yan, D.; Liu, G.; Bai, R.; Wang, D.; Chen, L.; Zhu, H.; An, H.; Kew, O.; Xu, W., Natural type 3/type 2 intertypic vaccine-related poliovirus recombinants with the first crossover sites within the VP1 capsid coding region. *PLoS ONE* **2010,** 5, (12), e15300.

10. Pliaka, V.; Filliponi, M. E.; Kyriakopoulou, Z.; Ruether, I. G.; Tsakogiannis, D.; Gartzonika, C.; Levidiotou-Stefanou, S.; Markoulatos, P., Retrospective molecular and phenotypic analysis of poliovirus vaccine strains isolated in Greece. *Clinical microbiology and infection : the official publication of the European Society of Clinical Microbiology and Infectious Diseases* **2011,** 17, (10), 1554-62.

11. Shahmahmoodi, S.; Parvaneh, N.; Burns, C.; Asghar, H.; Mamishi, S.; Tabatabaie, H.; Chen, Q.; Teimourian, S.; Gooya, M. M.; Esteghamati, A.-R.; Mousavi, T.; Yousefi, M.; Farrokhi, K.; Mashlool, M.; Kew, O.; Nategh, R., Isolation of a type 3 vaccine-derived poliovirus (VDPV) from an Iranian child with X-linked agammaglobulinemia. *Virus Research* **2008,** 137, 168-172.

12. Dedepsidis, E.; Pliaka, V.; Kyriakopoulou, Z.; Brakoulias, C.; Levidiotou-Stefanou, S.; Pratti, A.; Mamuris, Z.; Markoulatos, P., Complete genomic characterization of an intertypic Sabin 3/Sabin 2 capsid recombinant. *FEMS immunology and medical microbiology* **2008,** 52, (3), 343-51.

13. Savolainen-Kopra, C.; Samoilovich, E.; Kahelin, H.; Hiekka, A. K.; Hovi, T.; Roivainen, M., Comparison of poliovirus recombinants: accumulation of point mutations provides further advantages. *The Journal of general virology* **2009,** 90, (Pt 8), 1859-68.

14. Blomqvist, S.; Bruu, A. L.; Stenvik, M.; Hovi, T., Characterization of a recombinant type 3/type 2 poliovirus isolated from a healthy vaccinee and containing a chimeric capsid protein VP1. *Journal of General Virology* **2003,** 84, 573-580.

15. Tao, Z.; Wang, H.; Xu, A.; Zhang, Y.; Song, L.; Zhu, S.; Li, Y.; Yan, D.; Liu, G.; Yoshida, H.; Liu, Y.; Feng, L.; Chosa, T.; Xu, W., Isolation of a recombinant type 3/type 2 poliovirus with a chimeric capsid VP1 from sewage in Shandong, China. *Virus Res* **2010,** 150, (1-2), 56-60.
