# Supplementary material for: Pressure for Pattern-Specific Intertypic Recombination between Sabin Polioviruses: Evolutionary Implications
Source: Viruses. 2017 Nov 22;9(11):353. doi: 10.3390/v9110353 (PMC5707560; doi:10.3390/v9110353)
Supplement: Supplementary file 1 [file viruses-09-00353-s001.zip › Table S4.docx]

# Table S4. Recombinants whose partial genome sequences were reported previously

| # | **Lab. # of isolate** | **Source** | **Age or % nucleotide substitutions in VP1** | **Recombinant structure** | **Primary method of analysis** | **Reference** |
| --- | --- | --- | --- | --- | --- | --- |
| **Serotype 1** | | | | | | |
| 1 | 742 | sewage |  | S1-S3-S2 | RT-PCR screening | [[1](#_ENREF_1)] |
| **Serotype 2** | | | | | | |
| 2 | I 34 | AFP | ~7 weeks | S2-S1-S2-S1 | RFLP | [[2](#_ENREF_2)] |
| 3 | M-ov | AFP |  | S2-S1 | fingerprint | [[3](#_ENREF_3)] |
| 4 | Ts-os | AFP |  | S2-S1 | fingerprint | [[3](#_ENREF_3)] |
| 5 | V-ov | AFP |  | S2-S3 | fingerprint | [[3](#_ENREF_3)] |
| 6 | Zh-ch | AFP |  | S2-S1 | fingerprint | [[3](#_ENREF_3)] |
| 7 | Z-ya | AFP |  | S2-S3 | fingerprint | [[3](#_ENREF_3)] |
| 8 | 212 | AFP |  | S2-S1 | RFLP | [[4](#_ENREF_4)] |
| 9 | EP9 | AFP |  | S2-S1 | RFLP | [[5](#_ENREF_5)] |
| 10 | EP6 | AFP |  | S2-S1 | RFLP | [[5](#_ENREF_5)] |
| 11 | 2-IIIs12 | AFP |  | S2-S1 | RFLP | [[6](#_ENREF_6)] |
| 12 | 2-IIIs02 | AFP |  | S2-S3 | RFLP | [[6](#_ENREF_6)] |
| 13 | 2-IVs113 | AFP |  | S2-S1 | RFLP | [[6](#_ENREF_6)] |
| 14 | 2-IVn3 | AFP |  | S2-S1 | RFLP | [[6](#_ENREF_6)] |
| 15 | 2-IVs13 | AFP |  | S2-S1 | RFLP | [[6](#_ENREF_6)] |
| 16 | 151 | Healthy |  | S2-S1-S3 | fingerprint | [[7](#_ENREF_7)] |
| 17 | IF | Healthy |  | S2-S1 | RFLP | [[8](#_ENREF_8)] |
| 18 | EP12 | Healthy |  | S2-S1 | RFLP | [[5](#_ENREF_5)] |
| 19 | ENP5 | sewage |  | S2-S1 | RFLP | [[9](#_ENREF_9)] |
| 20 | ENP6 | sewage |  | S2-S1 | RFLP | [[9](#_ENREF_9)] |
| 21 | ENP7 | sewage |  | S2-S3 | RFLP | [[9](#_ENREF_9)] |
| 22 | ENP8 | sewage |  | S2-S1 | RFLP | [[9](#_ENREF_9)] |
| 23 | 155 | Unknown |  | S2-S1 | fingerprint | [[7](#_ENREF_7)] |
| 24 | 185 | Unknown |  | S2-S1 | fingerprint | [[7](#_ENREF_7)] |
| 25 | II-867 | Unknown |  | S2-S1 | fingerprint | [[7](#_ENREF_7)] |
| 26 | 6324A | Unknown |  | S2-S1-S3 | fingerprint | [[7](#_ENREF_7)] |
| 27 | 6324B | Unknown |  | S2-S3 | fingerprint | [[7](#_ENREF_7)] |
| **Serotype 3** | | | | | | |
| 28 | 30/79 | AFP | 2 weeks | S3-S1-S3 | fingerprint | [[10](#_ENREF_10)] |
| 29 | 6479/65 | AFP | 2 weeks | S3-S1-S3 | fingerprint | [[10](#_ENREF_10)] |
| 30 | IM | AFP |  | S3-S2-S1 | RFLP | [[8](#_ENREF_8)] |
| 31 | EP23 | AFP |  | S3-S1 | RFLP | [[5](#_ENREF_5)] |
| 32 | EPC | AFP |  | S3-S2-S3 | RFLP | [[11](#_ENREF_11)] |
| 33 | EDP12 | AFP |  | S3-S2-S1 | RFLP | [[11](#_ENREF_11)] |
| 34 | KT1-11/III | Healthy | <3 weeks | S3-S1 | fingerprint | [[12](#_ENREF_12)] |
| 35 | KT2-28/III | Healthy | <3 weeks | S3-S2-S3 | fingerprint | [[12](#_ENREF_12)] |
| 36 | KT2-10/III | Healthy | <3 weeks | S3-S2 | fingerprint | [[12](#_ENREF_12)] |
| 37 | EJM-47/III | Healthy | <3 weeks | S3-S2-S3 | fingerprint | [[12](#_ENREF_12)] |
| 38 | EJM-17/III | Healthy | <3 weeks | S3-S2 | fingerprint | [[12](#_ENREF_12)] |
| 39 | B7/15 | Healthy | 4 weeks | S3-S2-S3-S2-S3-S2-S3 | fingerprint | [[10](#_ENREF_10)] |
| 40 | KW7 | Healthy | <5 weeks | S3-S2-S3 | fingerprint | [[13](#_ENREF_13)] |
| 41 | KW5 | Healthy | <5 weeks | S3-S2 | fingerprint | [[13](#_ENREF_13)] |
| 42 | 66 | Healthy | <6 weeks | S3-S2-S3 | fingerprint | [[14](#_ENREF_14)] |
| 43 | 35 | Healthy | <6 weeks | S3-S2 | fingerprint | [[14](#_ENREF_14)] |
| 44 | 0223/84 | Healthy | 6 weeks | S3-S1 | fingerprint | [[10](#_ENREF_10)] |
| 45 | EP16 | Healthy |  | S3-S2 | RFLP | [[5](#_ENREF_5)] |
| 46 | EPA | Healthy |  | S3-S2-S3 | RFLP | [[11](#_ENREF_11)] |
| 47 | EGY/05/E5/K274 | sewage | 0.33 | S3-S2 | partial sequencing | [[15](#_ENREF_15)] |
| 48 | EGY/07/E87/V1 | sewage | 0.33 | S3-S2 | partial sequencing | [[15](#_ENREF_15)] |
| 49 | LK3 | sewage |  | S3-S2-S1 | RFLP | [[9](#_ENREF_9)] |
| 50 | LK6 | sewage |  | S3-S2-S1 | RFLP | [[9](#_ENREF_9)] |
| 51 | 584 | sewage |  | S3-S2-S1 | RT-PCR screening | [[1](#_ENREF_1)] |
| 52 | 738 | sewage |  | S3-S2-S1 | RT-PCR screening | [[1](#_ENREF_1)] |
| 53 | 106 | Unknown |  | S3-S2-S1 | fingerprint | [[7](#_ENREF_7)] |
| 54 | 115 | Unknown |  | S3-S2-S3 | fingerprint | [[7](#_ENREF_7)] |
| 55 | 116 | Unknown |  | S3-S2-S1 | fingerprint | [[7](#_ENREF_7)] |
| 56 | 156 | Unknown |  | S3-S2-S1 | fingerprint | [[7](#_ENREF_7)] |
| 57 | 158 | Unknown |  | S3-S2-S1 | fingerprint | [[7](#_ENREF_7)] |
| 58 | 6423 | Unknown |  | S3-S2-S1 | fingerprint | [[7](#_ENREF_7)] |

**References**

1. Pliaka, V.; Dedepsidis, E.; Kyriakopoulou, Z.; Mpirli, K.; Tsakogiannis, D.; Pratti, A.; Levidiotou-Stefanou, S.; Markoulatos, P., A new RT-PCR assay for the identification of the predominant recombination types in 2C and 3D genomic regions of vaccine-derived poliovirus strains. *Molecular and cellular probes* **2010,** 24, (3), 115-23.

2. Karakasiliotis, I.; Paximadi, E.; Markoulatos, P., Evolution of a rare vaccine-derived multirecombinant poliovirus. *The Journal of general virology* **2005,** 86, (Pt 11), 3137-42.

3. Lipskaya, G. Y.; Muzychenko, A. R.; Kutitova, O. K.; Maslova, S. V.; Equestre, M.; Drozdov, S. G.; Bercoff, R. P.; Agol, V. I., Frequent isolation of intertypic poliovirus recombinants with serotype 2 specificity from vaccine-associated polio cases. *J Med Virol* **1991,** 35, (4), 290-6.

4. Furione, M.; Guillot, S.; Otelea, D.; Balanant, J.; Candrea, A.; Crainic, R., Polioviruses with natural recombinant genomes isolated from vaccine-associated poliomyelitis. *Virology* **1993,** 196, 199-208.

5. Paximadi, E.; Karakasiliotis, I.; Mamuris, Z.; Stathopoulos, C.; Krikelis, V.; Markoulatos, P., Genomic analysis of recombinant sabin clinical isolates. *Virus genes* **2006,** 32, (2), 203-10.

6. Georgescu, M. M.; Balanant, J.; Ozden, S.; Crainic, R., Random selection: a model for poliovirus infection of the central nervous system. *The Journal of general virology* **1997,** 78 ( Pt 8), 1819-28.

7. King, A. M., Genetic recombination in positive strand RNA viruses. In *RNA Genetics*, Domingo, E.; Holland, J. J.; Ahlquist, P., Eds. CRC-Press: University of Michigan, 1988; pp 149-165.

8. Karakasiliotis, I.; Markoulatos, P.; Katsorchis, T., Site analysis of recombinant and mutant poliovirus isolates of Sabin origin from patients and from vaccinees. *Molecular and cellular probes* **2004,** 18, (2), 103-9.

9. Paximadi, E.; Karakasiliotis, I.; Papaventsis, D.; Papageorgiou, G.; Markoulatos, P., Recombinant Sabin environmental isolates in Greece and Cyprus. *J Appl Microbiol* **2008,** 104, (4), 1153-62.

10. Driesel, G.; Diedrich, S.; Kunkel, U.; Schreier, E., Vaccine-associated cases of poliomyelitis over a 30 year period in East Germany. *European Journal of Epidemiology* **1995,** 11, (6), 647-654.

11. Paximadi, E.; Karakasiliotis, I.; Bolanaki, E.; Krikelis, A.; Markoulatos, P., Vaccine derived bi- and multi-recombinant Sabin strains. *Virus genes* **2007,** 35, (3), 541-8.

12. Cammack, N.; Phillips, A.; Dunn, G.; Patel, V.; Minor, P. D., Intertypic genomic rearrangements of poliovirus strains in vaccinees. *Virology* **1988,** 167, 507-514.

13. Tatem, J.; Weeks-Levy, C.; Mento, S. J.; Di Michele, S. J.; Georgiu, A.; Waterfield, W. F.; Sheip, B.; Costalas, C.; Davies, T.; Ritchey, M. B.; Cano, F. R., Oral poliovirus vaccine in the United States: molecular characterization of Sabin type 3 after replication in the gut of vaccinees. *Journal of Medical Virology* **1991,** 35, 101-109.

14. Minor, P. D.; John, A.; Ferguson, M.; Icenogle, J. P., Antigenic and molecular evolution of the vaccine strain of type 3 poliovirus during the period of excretion by a primary vaccinee. *Journal of General Virology* **1986,** 67, (4), 693-706.

15. Blomqvist, S.; Savolainen-Kopra, C.; Paananen, A.; El Bassioni, L.; El Maamoon Nasr, E. M.; Firstova, L.; Zamiatina, N.; Kutateladze, T.; Roivainen, M., Recurrent isolation of poliovirus 3 strains with chimeric capsid protein Vp1 suggests a recombination hot-spot site in Vp1. *Virus Res* **2010,** 151, (2), 246-51.
